# A Test of Rensch’s Rule in Greater Horseshoe Bat (*Rhinolophus ferrumequinum*) with Female-Biased Sexual Size Dimorphism

**DOI:** 10.1371/journal.pone.0086085

**Published:** 2014-01-20

**Authors:** Hui Wu, Tinglei Jiang, Xiaobin Huang, Hongjun Lin, Hongwei Wang, Lei Wang, Hongxing Niu, Jiang Feng

**Affiliations:** 1 Jilin Key Laboratory of Animal Resource Conservation and Utilization, Northeast Normal University, Changchu, China; 2 Key Laboratory for Wetland Ecology and Vegetation Restoration of National Environmental Protection, Northeast Normal University, Changchun, China; 3 College of Life Sciences, Henan Normal University, Xinxiang, Henan, China; University of Florence, Italy

## Abstract

Sexual size dimorphism (SSD) is widespread within the animal kingdom. Rensch’s rule describes a relationship between SSD and body size: SSD increases with body size when males are the larger sex, and decreases with body size when females are the larger sex. Rensch’s rule is well supported for taxa that exhibit male-biased SSD but patterns of allometry among taxa with female-biased size dimorphism are mixed, there is evidence both for and against the rule. Furthermore, most studies have investigated Rensch’s rule across a variety of taxa; but among-population studies supporting Rensch’s rule are lacking, especially in taxa that display only slight SSD. Here, we tested whether patterns of intraspecific variation in SSD in greater horseshoe bats conform to Rensch’s rule, and evaluated the contribution of latitude to Rensch’s rule. Our results showed SSD was consistently female-biased in greater horseshoe bats, although female body size was only slightly larger than male body size. The slope of major axis regression of log_10_ (male) on log_10_ (female) was significantly different from 1. Forearm length for both sexes of greater horseshoe bats was significantly negatively correlated with latitude, and males displayed a slightly but nonsignificant steeper latitudinal cline in body size than females. We suggest that variation in patterns of SSD among greater horseshoe bat populations is consistent with Rensch’s rule indicating that males were the more variable sex. Males did not have a steeper body size–latitude relationship than females suggesting that sex-specific latitudinal variation in body size may not be an important contributing factor to Rensch’s rule. Future research on greater horseshoe bats might best focus on more comprehensive mechanisms driving the pattern of female-biased SSD variation.

## Introduction

Sexual size dimorphism (SSD) is commonplace within the animal kingdom [1]. SSD may be absent (monomorphism), male-biased, or female-biased. Since the 1960s, sexual size dimorphism has been the focus of a number of studies [2]–[4]. One primary objective is to test whether the pattern of SSD variation conforms to Rensch’s rule which predicts that when males are larger than females, SSD increases with increasing body size, but when females are larger than males, SSD decreases in larger species. In other words, Rensch’s rule predicts that male size varies more than female size, and male is purportedly the driver of size divergence whereas female size co-varies passively with that of males, thereby generating a pattern of allometry in SSD [5], [6]. Rensch’s rule has been confirmed by observations in various animal taxa including insects, fishes, reptiles, birds and mammals [3], [7]–[12]. However, Rensch’s rule does not apply universally; while it is well supported for taxa that exhibit male-biased SSD or mixed SSD ([2], [3], [8], [10], for the exception see [13]), patterns of allometry among taxa with female-biased size dimorphism are less clear and there is evidence both for [11], [14], [15] and against [4], [16] the rule.

Rensch’s rule was originally formulated at the interspecific level and most publications on SSD present broad interspecific comparisons [2], [17], [18]. Investigations of intraspecific variation in SSD patterns, in contrast, are less common [19]. So far, it remains unclear whether within-species variation in SSD supports Rensch’s rule, especially for taxa with female-biased size dimorphism.

Rensch’s rule is traditionally explained by sexual selection or/and natural selection, with sexual selection being either inter-sexual (epigamic selection) or intra-sexual [20], [21]. Sexual selection currently appears to be the most likely mechanism, at least in mammals, birds and reptiles [8], [22]. Females facing weak or antagonistic selection and males experiencing strong sexual selection could explain why male size varies more than female size, as predicted by Rensch’s rule. For example, sexual selection favors larger males in species that display male-biased SSD, because larger males accrue greater reproductive success [1]. In contrast, in taxa that display female-biased SSD, sexual selection favors smaller males because individuals with smaller body size are more agile and are superior in scramble competition [1], [23] or aerial courtship displays (agility hypothesis) [24]–[27]. Intraspecific patterns of SSD that support Rensch’s rule can be explained by genetic adaptation to the locally occurring strength of sexual selection on males [28]. These patterns may, however, also be a product of males having greater phenotypic plasticity in body size than females [29]. Additionally, sex-specific latitudinal variation is an important contributing factor to Rensch’s rule at the intraspecific level, or in other words, male body size is more variable with latitude than female body size [19].

The majority of mammals exhibit sexual size dimorphism, and the most of them display male-biased size dimorphism [5], [30]. Bats, however, primarily display female-biased sexual size dimorphism [31]. Previous studies found that many species of Vespertilionidae, Rhinolophidae and Pteropodidae exhibit female-biased SSD in both body mass and skeletal measures (e.g. forearm length) [32]–[35], but these studies focused mostly on a description of SSD or clinal variation of SSD [35]. Moreover, one study demonstrated that SSD of vespertilionid bats is associated with litter size per pregnancy [33], although another study analyzing different species was unable to corroborate this result [36]. Therefore, to our knowledge, little is known about whether bats exhibit Rensch’s rule at the intraspecific or interspecific level or what may cause different SSD.

Greater horseshoe bats (*Rhinolophus ferrumequinum*) provide a unique opportunity to test Rensch’s rule. *R. ferrumequinum* has a wide range in the Palaearctic [37] and is one of the most widespread bat species in China [38]. Moreover, studies have determined the presence of reverse SSD in both body mass and forearm length [34], [39]. Additionally, a study recently demonstrated that male greater horseshoe bats can experience strong sexual selection, but it seems that sexual selection among males operates independently of body size [40]. In this present study, we provide the first intraspecific tests of Rensch’s rule in greater horseshoe bats using data from 23 populations in a broad geographic area. Our objectives were: (1) to test whether patterns of intraspecific variation in SSD in greater horseshoe bats conform to Rensch’s rule, and (2) to evaluate the contribution of natural selection to Rensch’s rule by assessing the relationship between latitude and SSD.

## Materials and Methods

### Ethics Statement

All experimental procedures were in accordance with the relevant guidelines and regulations for experiments involving vertebrate animals of the People’s Republic of China, and were approved by Jilin Wildlife Conservation Council, Department of Forestry of Jilin Province (Permit Number: [2006] 178). Experiments were carried out under an Institutional Animal Care and Use Protocol of Northeast Normal University that approved this study (Permit Number: NENU-W-2008-108). All animals were handled gently during measurements of body size. After the data collection, all animals were released in the same cave where they were initially collected from.

### Data Collection

We examined morphological data on the body size from 23 populations in *R. ferrumequinum*, 10 populations came from published literature and 13 populations from our data set (see Table 1 and Table S1 for details). Although body mass might seem the gold standard for estimating overall size, mammalian weights are subject to many sources of variation which often make them less reliable than linear measurements such as skeletal measures [41]. Here, we used forearm length (FA) as a proxy for body size for the following reasons. First, in bats, body mass exhibits poor repeatability within individuals because of effects of different amounts of food within intestinal system, seasonal differences in weight, and reproductive condition (especially in females) [42]. Second, forearm length could be measured relative easily and accurately in live bats in the field. Third, forearm length in bats is the most common measure for body size, including studies on SSD [32]–[35], [42], [43]. Fourth, one may expect females to have proportionately larger wings than males in order to offset additional weight caused by fetus and pup carrying, resulting in a possible bias of our measurements of overall body size. When using head body length (HBL) as a covariate to adjust forearm length, however, in most bat species, the adjusted forearm length of females was virtually identical to that of males [36]. Finally, experiments in non-pregnant females with offspring-sized weights appropriately attached showed that bats increase their effective airfoil area by making postural adjustments during flight to compensate for any additional mass, suggesting this may also occur naturally in pregnant bats carrying additional mass caused by the offspring as gestation progresses [44], [45]. More recently, a study determined that females actually depended on different wing shapes rather than different wing sizes to compensate for any additional weight caused by carrying a fetus during pregnancy or a newborn [46].

**Table 1:** Location, sample size, and mean forearm length in males and females of 23 populations of *Rhinolophus ferrumequinum*.

Sites	location			sample size	female forearm length (mm)		male forearm length (mm)		References
	latitude	longtitude	altitude	female/male	mean±SD	min–max	mean±SD	min–max	
Ji’an, China	41°3′ N	125°50′E	250	77/31	59.46±0.91	57.13–61.54	58.28±1.47	53.61–60.02	Unpublished own data
Luotongshan, China	41°49′N	126°10′E	553	6/6	58.67±1.63	57.00–61.00	58.5±1.52	57.00–61.00	Unpublished own data
Yongji, China	43°28′N	125°56′E	377	10/12	58.92±0.88	57.39–60.2	58.32±1.33	56.58–60.47	Unpublished own data
An’shan, China	41°21′N	124°54′E	524	5/5	59.93±0.80	58.54–60.69	58.71±1.68	56.83–60.37	Unpublished own data
Jinning, China	24°29′N	102°22′E	2202	12/16	61.08±2.16	55.00–63.50	60.34±1.42	57–62	Unpublished own data
Dali, China	25°34′N	100°13′E	2351	4/4	60.88±3.84	56.00–65.00	60.88±0.63	60.00–61.50	Unpublished own data
Zibo, China	36°16′N	118°04′E	570	11/12	58.32±1.34	55.95–60.37	57.95±1.16	55.92–59.64	Unpublished own data
Baoji, China	35°02′N	106°40′E	1489	32/17	59.50±1.52	55.74–63.27	59.12±1.37	55.39–61.45	Unpublished own data
Xi’an, China	34°04′N	109°24′E	897	15/6	59.17±1.67	56.50–62.32	58.2±2.28	55.53–61.73	Unpublished own data
Shangluo, China	33°35′N	109°09′E	715	7/8	60.69±0.75	60.00–61.85	59.25±1.43	56.78–61	Unpublished own data
Nanyang, China	32°23′N	113°16′E	589	22/7	59.5±0.88	57.70–61.35	58.19±2.18	55.39–61	Unpublished own data
Tianshui, China	34°20′N	106° 00′E	1812	8/11	59.23±0.71	58.44–60.00	57.52±1.87	54.81–59.74	Unpublished own data
Beijing, China	39°42′N	115°43′E	516	7/6	59.88±0.82	59.00–61.00	59.05±1.03	57.01–60.00	Unpublished own data
Mentougou, China	39°56′N	116°06′ E	234	5/6	59.8±0.45	59.00–60.00	59±1.79	57.00–62.00	Wu et al.(2009)
Huanren, China^a^	41°21′N	124°54′E	524	5/5	58.5	55.80–60.00	56.4	48.00–59.20	Xiao et al.(1988)
Greece and Turkey^a^				117/1010	58.30±1.28	53.70–62.40	57.0±1.37	53.00–60.50	Christian Dietz. et.al (2006)
Syria				20/6	58.42±1.98	54.30–61.20	56.02±2.23	52.80–58.20	Benda P. et al.(2006)
Cyprus				6/5	56.40±1.57	54.80–58.00	55.7±1.26	54.6–57.7	Benda P. et al.(2007)
Jodan				4/6	58.28±1.96	56.60–61.10	57.67±0.83	57.10–58.80	Benda P. et al.(2011)
Irans				31/38	57.51±2.07	55.00–61.00	56.2±2.09	52.50–60.00	DeBlase A. F. et al(1980)
Tsushima andNagasaki^a^	34°14′N	129°17′E	298	19/6	59.18	56.62–61.65	58.02	56.53–59.30	Kuniko Kawa. et al. (2007)
India				6/3	57.08±2.06	54.00–60.00	54.33±1.89	53.00–56.50	Sinha,Y.P. (1973)
Como, Sondrio^a^				3/7	57.25±0.53		56.09±1.09		Peratoni D. et al.(2000)

^a^ Denotes populations without body size data for individuals.

From the literature we collected data on forearm length of greater horseshoe bats captured at various places in Meng tougou, Hebei, China [47], Huanren, Liaoning, China [48], Bulgaria, Greece, and western Turkey [34], Syria [49], Cyprus [50], Jordan [51], Iran [52], Japan [53], India [54], and Italy (Como and Sondrio) [55]. Forearm length of different individuals was available from 7 sites and mean forearm length was available for 3 other sites. All data were taken only from adults, and at least five males and five females were measured at each site. Unfortunately, 7 of 10 sites did not provide accurate latitude data (see Table 1 for details).

For our own data collection, we captured bats between 2008 and 2013 from thirteen roosts occupied by *R. ferrumequinum* in 9 provinces of China. Bats were captured with mist nets when leaving or entering cave day-roosts or with hand nets inside the caves. They were kept individually in cloth bags until measured. Bats were sexed and classified as juvenile or adult. The age class of the bat was determined by the degree of closure of epiphyseal growth plates of phalanges and by comparing fur coloration and structure with banded bats of known ages [56], [57]. In this study, only adult bats were measured. Forearm length was measured to the nearest 0.01 mm with electronic calipers (TESA-CAL IP67, Switzerland). To standardize potential errors, each measurement was made three times, and mean values were used in the subsequent analysis. For each roost site, we also determined its latitude, longitude and elevation using GPS (eTrex Vista, Garmin International Inc., Olathe, KS, USA). Every individual was kept in the bags for approximately ½ hour. All individuals were subsequently released at the site of capture.

### Testing Rensch’rule

Forearm length was log_10_-transformed to meet assumptions of a normal distribution. Sex differences in FA among populations were tested using a two-way factorial ANCOVA with log_10_ (FA) as the dependent variable, latitude as the covariate and sex and population as fixed-effects (14 populations and 368 individuals were included in this analyses. 9 additional populations were excluded because we lacked either data from individuals or accurate latitude data). The factorial design provided tests of four null hypotheses: after controlling for the correlated effects of the latitude on forearm length (1) no effect of geographic locality, (2) no sexual dimorphism, (3) no geographic variation in sexual dimorphism (as indicated by population × sex interaction), and (4) the regression slope for predicting forearm length from latitude is the same in female and male groups (as indicated by latitude × sex interaction).

A standard approach, which we followed to test for Rensch’s rule, is to test whether a bivariate plot of log_10_ female size versus log_10_ male size has a slope significantly different from 1.0 (i.e. exhibits isometry) [5], [58]. When male size is plotted on the x-axis, a slope significantly less than 1.0 or when female size is plotted on the x-axis, a slope significantly more than 1.0 provides evidence for Rensch’s rule [5]. Both conditions suggest a greater variance in male than female body size (Δmale size>Δfemale size) regardless of which sex is plotted on the x-axis. We opted to follow Fairbairn (1997) and plotted female forearm length on the x-axis [5]. When performing a normal regression of male and female forearm length, measurement errors will be approximately equal in both sexes. Therefore, we used major axis regression to estimate the slopes of the linear regression and tested the null hypothesis of b = 1 using 95% confidence intervals. We used the Smatr R package [59] for these analyses.

To examine the relationship between forearm length of males and females and latitude, respectively, we used Model I linear regression. To determine if the slopes of the regression lines for males and females were significantly different at the population level, we applied an Analysis of Covariance (ANCOVA). The mean forearm length of each population was log_10_-transformed and used as dependent variable, latitude as a covariate, and sex as a fixed-factor. Finally, linear and quadratic regression determined the relationship between SSD (log_10_(female size) – log_10_(male size)) and latitude. Only 16 populations of the entire data set obtained from the literature and from our own data pool, however, provided accurate latitude data and were therefore included in this analysis. Nevertheless, these populations covered a wide range of latitudes ranging from 24°29′N to 43°28′N, virtually including the entire range of latitudes examined in all studies.

All analyses were performed using R statistical language [60].

## Results

Our results showed that SSD was consistently female-biased in greater horseshoe bats, with females being slightly larger than males (Table 1). Overall, the mean ± SD forearm length of females was 59.098±1.73 (n = 288) and that of males 58.10±2.06 (n = 195). Univariate ANCOVA of forearm length revealed a significant effect of latitude on forearm length (*F_1,340_* = 27.99, *P*<0.001), a high degree of heterogeneity among populations (*F_12,340_* = 4.17, *P*<0.001), and a significant difference between the sexes (*F_1,340_* = 34.70, *P*<0.001). However, sex differences were not statistically significant among populations (*F_12,340_* = 0.71, *P* = 0.73), and the regression slope of forearm length and latitude in females was not significantly different from the slope in males (*F_1,340_* = 0.23, *P = *0.63; Table 2).

**Table 2:** Effects of geographic sites and sex on variation in forearm length of *Rhinolophus ferrumequinum* as revealed by an univariate two-way ANCOVA with latitude as covariate.

Effect	Log_10_ (forearm length)				
	df	SS	MS	*F*	*P*
Latitude	1	0.00289	0.003	27.99	<0.001
Population	12	0.00516	0.0004	4.17	<0.001
Sex	1	0.00358	0.0036	34.70	<0.001
Latitude × Sex	1	0.00002	0.00002	0.23	0.63
Population × Sex	12	0.00088	0.000074	0.71	073
Residuals	340	0.03507	0.000103		

This analysis is based on length of forearm for the 14 sites (n = 368).

df, degrees of freedom; SS, sum of squares; MS, mean square.

**Figure 1:**
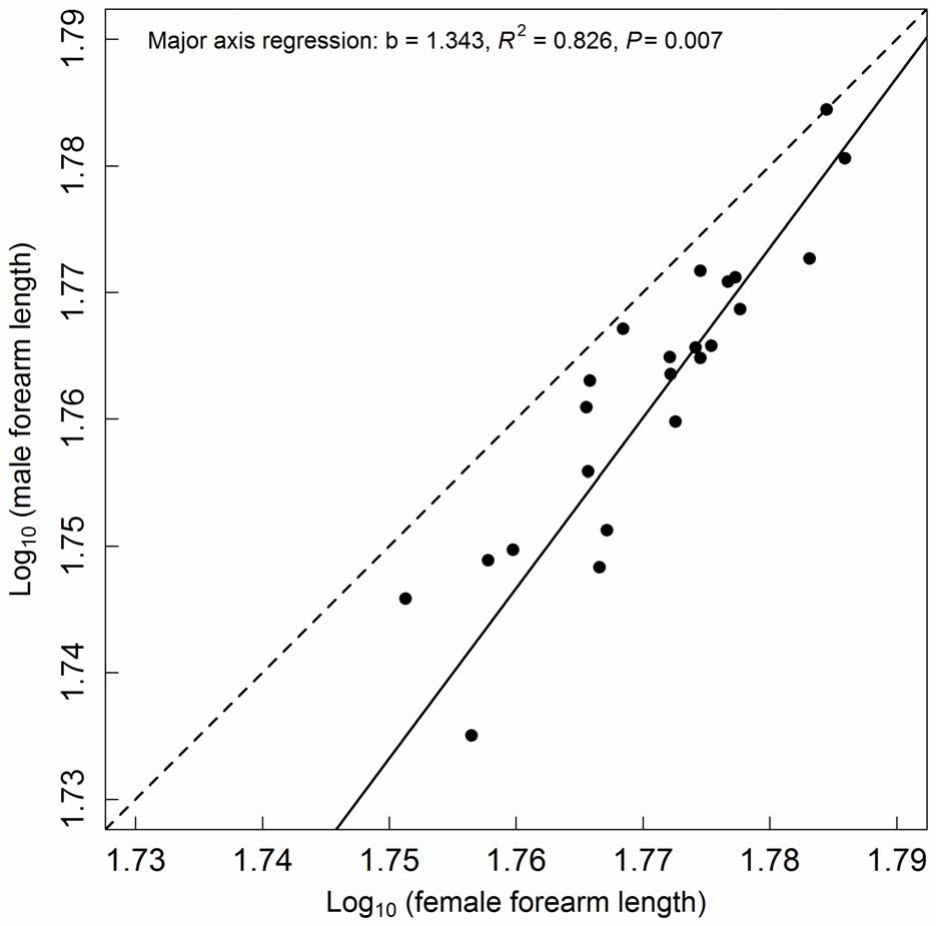
Rensch’s rule in the greater horseshoe bat, *Rhinolophus ferrumequinum.* Log_10_ (mean male forearm length) is plotted against log_10_ (mean female forearm length). The dashed line represents isometry, the solid line represents major axis linear regression line (slope = 1.343). Each dot represents a single population based on the mean forearm length of females and males (n = 23).

Although the magnitude of SSD that we found is modest, the variation pattern in SSD among different greater horseshoe bat populations was consistent with Rensch’s rule, with male size being more variable than female size. The slope of the major axis regression of log_10_ (male) on log_10_ (female) was significantly different from 1 (*R^2^* = 0.826, slope = 1.343, intercept = −0.62, 95%CI = 1.094–1.670, *P* = 0.007; Fig. 1).

**Figure 2:**
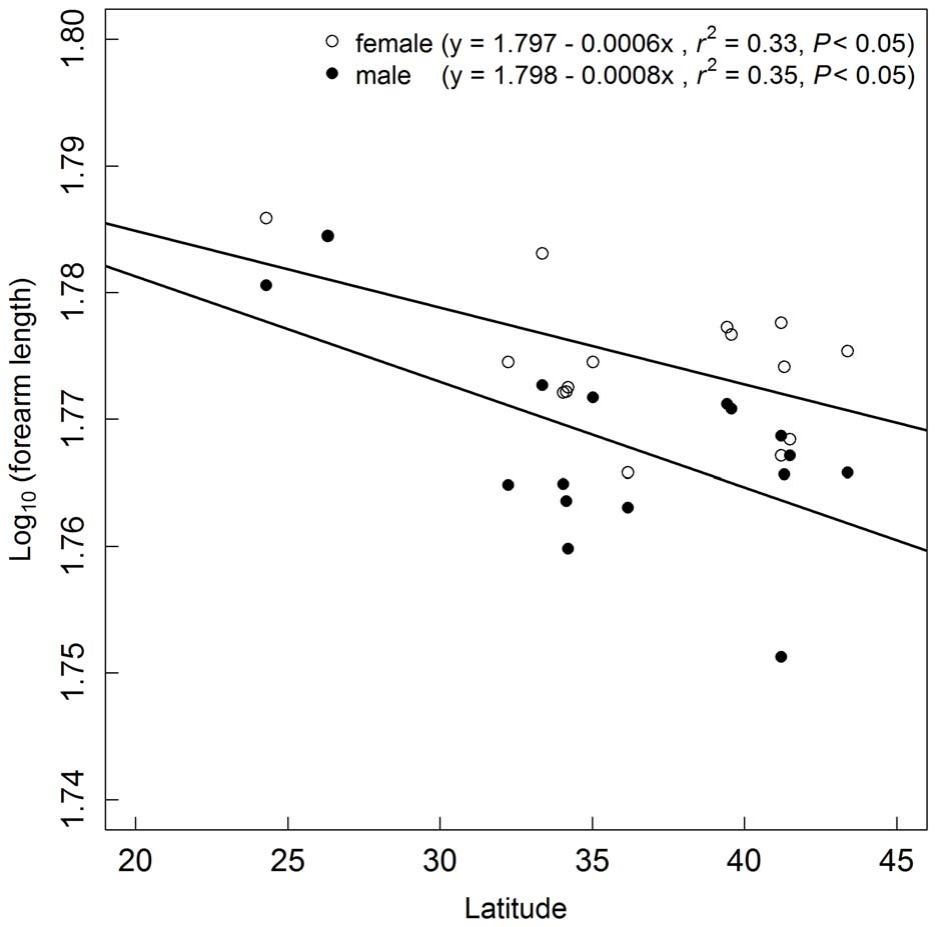
Model I linear regression of mean forearm length against latitude of females and males (n = 16).

In order to explain Rensch’s rule, we tested the contribution of latitude, which was used as a proxy for environmental conditions. We examined if body size varied more with latitude in males than in females. We found that forearm length for both sexes was significantly negatively correlated with latitude (female: *r^2^* = 0.33, *p*<0.05; male: *r^2^* = 0.35, *p*<0.05). There was a slight trend toward steeper body size–latitude relationships in males (slope = 0.0008) than in females (slope = 0.0006)(Fig. 2). Nevertheless, ANCOVA results at the population level revealed slopes of regression lines for males and females were not significantly different (*F_1,28_* = 0.349, *P* = 0.559, Table 3) despite having different intercepts. It appears that greater horseshoe bats were more dimorphic at high latitudes, but regression results suggested latitude was not significantly correlated with SSD (linear regression: *r^2^* = 0.029, *df* = 14, *p = *0.247; quadratic regression: *r^2^* = −0.014, *df* = 13, *p* = 0.433).

**Table 3:** Results of ANCOVA for testing homogeneity of slopes between latitude and forearm length in two sex groups.

Effect	Mean Forearm length (mm)				
	df	SS	MS	*F*	*P*
Latitude	1	0.00047	0.00047	14.225	<0.001
Sex	1	0.00042	0.00042	12.598	<0.01
Latitude×Sex	1	0.00001	0.000001	0.349	0.559
Residuals	28	0.0009	0.00003		

This analysis is based on mean forearm length from 16 populations.

## Discussion

Here, we used data from 23 populations to test whether the pattern of SSD variation in *R. ferrumequinum* with female-biased SSD conformed to Rensch’s rule and whether males and females displayed consistently different latitudinal clines in body size. Our results showed that the variation pattern in SSD among greater horseshoe bat populations was consistent with Rensch’s rule with male size being more variable than female size, but males did not vary more with latitude than females suggesting that sex-specific latitudinal variation in body size may not be an important contributing factor to Rensch’s rule.

Corroborating previous work [34], [40], our results indicated that *R. ferrumequinum* is a species with female-biased SSD in all populations. In addition, we provide the first intraspecific test of Rensch’s rule in any bat species, and also present a new example, unusual for a female-biased taxon, in which allometry for SSD is consistent with Rensch’s Rule. Other studies on systems with primarily female-biased SSD are rare [6], [15], [61]. It seems Rensch’s rule was not applied as a general phenomenon in taxa with female-biased SSD, but the reported patterns still follow Rensch’s rule in many cases [11], [14], [62], which is consistent with our own findings presented here.

The observed Rensch’s rule suggests male size varies more than female size (this can also be inferred from the larger standard deviation of forearm length in males compared with females, see results). Most available evidence supports the view that sexual selection on male body size is an important contributing factor to explain Rensch’s rule [8], [17], [28]. This is unlikely to be the case for greater horseshoe bats. Rossiter et al. (2006) showed that male greater horseshoe bats experienced as strong a sexual selection as males in male-dimorphic polygynous species. However the authors were unable to link the opportunity for sexual selection to selection on body size or any other particular male traits [40]. Therefore, in greater horseshoe bats, there is no evidence for a significant relationship between body size and sexual selection pressure on males, and hence we abstained from using sexual selection on males to explain Rensch’s rule in the present study.

Another contributing factor to Rensch’s rule may be natural selection. Our results showed that forearm length for both sexes was negatively correlated with latitude and male body size varied slightly more with latitude than female body size, although the slopes of the body size-latitude lines between sexes were not significantly different and SSD did not vary significantly with latitude. Our data therefore do not support the hypothesis that sex-specific latitudinal variation is a mediator of the intraspecific equivalent of Rensch’s rule. However, although there may indeed exists a correlation between SSD and latitude, the small differences in SSD found here may require even larger sample sizes as we used to unequivocally demonstrate statistical significance. Although our total sample size included 23 populations, only data from 16 populations included the required accurate latitude data and were therefore included in our analysis. Hence, in future studies, larger sample sizes and characterization of more ecological variables, such as food availability, population density, and interspecific interactions, need to be considered to test if any environmental factors contribute to Rensch’s rule.

Although Rensch’s rule may result from stronger selection pressure on males than females, this does not rule out any selection on females. The big-mother hypothesis [41] states that there could be some reason for this reversed SSD in mammals. In many vespertiolionid bat species, females are larger than males, and the degree of sexual size dimorphism correlates with litter size [33]. In great fruit-eating bats, wing elements that enhance aerodynamic performance were larger in females than in males, both in absolutely and relatively measures [63]. In our study, however, this was not the case because greater horseshoe bats produce normally only one young per litter [43] and our data are insufficient to demonstrate the presence or absence of larger wing areas in females, because forearm length alone is probably not a good index compared to total wing area, wing loading, or other morphological characteristics affecting the efficiency of their flight (see [64]). The longer forearms of female greater horseshoe bats most likely simply reflect their larger body size. Larger bats can fly faster and the cost of transport decreases with greater size [65]. Larger size may also permit females to carry a large quantity of insects in their intestinal system, and may allow them to produce more milk nurse their young.

Bergmann’s rule states that body size increases with increasing latitude (or colder climate) [66]. Our analysis of forearm length in greater horseshoe bats corroborates data using skull size of the same species in south-eastern Europe [67], indicating an inverse of Bergmann’s rule, a pattern not frequently observed in mammals (but see [68]). The resource restriction hypothesis states that body size correlates directly with the duration of resource availability, and that body size is a “function of how much time growing individuals have unhindered access to food of the highest quality” [69]. We found here that greater horseshoe bats from southern China (low latitude, such as the population in Dali with a latitude 24°29′N) undergo a short period of torpor during hibernation (about one month, Jiang Feng, personal observation), while those in northern China (high latitude, such as the population in Ji’an with a latitude 41°3′ N) remain in torpor for much longer during hibernation (about six months, Tinglei Jiang, personal observation over 6 years). Therefore, we suggest that greater horseshoe bats from colder areas are born later, start hibernating earlier, and thus have less time to access the food resources and complete their development than their conspecifics from more southerly regions. The resource restriction hypothesis may thus explain the negative Bergmann’s response in greater horseshoe bats.

In conclusion, our results suggested that greater horseshoe bats exhibited a slight female-biased SSD in forearm length, and that the variation pattern in SSD was consistent with Rensch’s rule. We also showed that latitude may not be an important contributing factor to explain Rensch’s rule at the intraspecific level. It remains a challenge to determine the reasons underlying Rensch’s rule in greater horseshoe bats. We suggest that future studies should focus on the following three aspects. From a life history point of view, they should test whether sex-specific growth and development contribute to Rensch’ rule. From an evolutionary/genetic point of view, future work should to examine whether more variation in male body size has a genetic basis. And finally, from an ecological/environmental point of view, more climatological and ecological variables should be considered to test if there are some environmental factors that affect one sex more than the other and thus result in Rensch’s rule.

## Supporting Information

Table S1 Summary of the raw data of forearm length of *Rhinolophus ferrumequinum* from our data set.
(XLSX)
